# Long-term non-invasive ventilation reduces readmissions in COPD patients with two or more episodes of acute hypercapnic respiratory failure

**DOI:** 10.3402/ecrj.v3.28303

**Published:** 2016-03-31

**Authors:** Kasper Linde Ankjærgaard, Sophia Liff Maibom, Jon Torgny Wilcke

**Affiliations:** Department of Pulmonary Medicine, Gentofte Hospital, Hellerup, Denmark

**Keywords:** COPD, ventilation, respiratory failure, hypercapnia, exacerbations

## Abstract

**Background:**

Chronic obstructive pulmonary disease (COPD) patients who have had an episode of acute hypercapnic respiratory failure (AHRF) have a large 1-year risk of death or readmission. Acute non-invasive ventilation (NIV) has been shown to be an effective treatment of AHRF; and long-term NIV (LTNIV) has been shown to be an effective treatment of chronic respiratory failure in stable hypercapnic COPD. We investigated the effects of LTNIV in a group of patients with severe, unstable COPD: frequent admissions and multiple previous episodes of AHRF treated with NIV.

**Methods:**

We conducted a retrospective analysis of 20 COPD patients treated with LTNIV after two or more episodes of AHRF during 1 year.

**Results:**

The mean number of AHRF episodes decreased from 2.44 in the year prior to LTNIV initiation to 0.44 in the year following (*p<*0.0001). The median number of admissions decreased from 5.19 to 1.88 (*p=*0.0092). Four patients (20%) died in 1 year. LTNIV tended to reduce arterial CO_2_. No changes were found in lung function.

**Conclusions:**

LTNIV seems effective in reducing recurrent AHRF and readmissions in a highly select group of patients with severe, unstable COPD and frequent AHRF.

In exacerbations of severe chronic obstructive pulmonary disease (COPD), patients are at risk of developing acute hypercapnic respiratory failure (AHRF). Patients with AHRF due to COPD necessarily have to be admitted for treatment. In accordance with guidelines from the Global Initiative for Chronic Obstructive Lung Disease (GOLD), non-invasive positive pressure ventilation (NIV) is recommended as a part of the first line treatment for AHRF due to COPD, along with oxygen supply and medical treatment (1–3).

Patients having survived an AHRF have a poor prognosis as they are highly susceptible to readmissions (79.9%), life-threatening events (63.3%), and death (49.1%) (4).

Long-term NIV (LTNIV) has been shown to reduce mortality in stable COPD patients (with no exacerbations for at least 4 weeks prior to enrollment) with chronic hypercapnic respiratory failure (CHRF) (5). LTNIV also has proven effective in chronic respiratory failure due to neuromuscular disorders (6).

It is hypothesized that LTNIV may prevent exacerbations and recurrent AHRF in selected COPD patients with frequent episodes of AHRF. Two small randomized controlled trials have shown a significantly longer time to worsening in patients treated with LTNIV and a lower risk of readmissions (7, 8). A non-randomized observational study showed reduced mortality (9).

Large randomized controlled trials (RCTs) showed improvements of arterial gases but no effects on survival or admissions in COPD patients treated with LTNIV after an AHRF (10, 11).

The purpose of this study was to investigate the effect of LTNIV initiated in COPD patients with two or more admissions due to AHRF within 1 year.

## Methods

This study was a retrospective analysis of patient journals, arterial gas samples, and lung function parameters. The study population consisted of COPD patients enrolled for LTNIV in the Department of Pulmonary Medicine at Gentofte Hospital from 2009 to 2014. LTNIV was offered to COPD patients who had had two or more episodes of AHRF treated with acute NIV within 1 year.

We defined AHRF as an exacerbation of COPD with a *P*_a,CO2_>6 kPa and an arterial pH≤7.35 despite 1–2 h of treatment with nebulized bronchodilators, intravenous methylprednisolone, and oxygen supply according to the GOLD recommendations (2).

The aim of the acute NIV treatment was to reverse acidosis and to normalize hypercapnia.

Patients were initiated on LTNIV after an AHRF – the second or more in 1 year. Patients did not have to be persistently hypercapnic at discharge. The home ventilator was adjusted to the settings and pressures that had reversed acidosis and reduced hypercapnia during the AHRF. Thus, LTNIV was a continuation of the acute NIV treatment.

The patients and their caregivers were taught to use, clean, and maintain the ventilator; they could contact a hotline run by nurses competent in NIV.

We recommended the use of LTNIV each night and during a daytime nap.

When COPD exacerbations occurred, patients were treated with steroids, antibiotics, and bronchodilators, according to the GOLD guidelines.

Patients with obstructive sleep apnea (OSA), obesity hypoventilation syndrome (OHS), or cancer were excluded from this analysis.

We reviewed each patient's journal from 1 year before LTNIV initiation to 1 year after.

We noted baseline data for all the patients initiated with LTNIV, denoted as *intention to treat*.

Patients who survived 1 year of LTNIV were denoted *per protocol* (PP).

For PP patients, we compared the following variables before LTNIV initiation to 1 year after: the number of episodes of AHRF treated with acute NIV per year; the number of admissions to a pulmonary ward; the number of days admitted due to COPD; lung function; use of long-term oxygen treatment (LTOT); and arterial gas values.

It was assumed that the clinical and physiological status of the patients would not improve spontaneously without intervention. Thus, the patients were their own controls, and any reduction in the numbers of AHRF episodes, admissions, or admission days could be attributed to the LTNIV treatment.

By visual analysis, we found that data followed an acceptable normal distribution. Accordingly, differences were analyzed using parametric tests: the Student's *t*-test and chi-square test.

We used SAS 9.4 (SAS Institute, Cary, NC, USA) for the statistical analyses.

### Ethics

No ethics committee approval was needed because the study was retrospective and no data contained personally identifiable information.

## Results

As of October 2015, 41 COPD patients had been treated with LTNIV for at least 1 year at the Department of Pulmonary Medicine of Gentofte Hospital. Of this number, 21 patients were not eligible for this analysis: 8 patients had OSA, 3 had OHS, and 1 had a combination of both. Five patients had not had two episodes of AHRF prior to LTNIV initiation. Two patients had initiated the LTNIV treatment a long time before being affiliated with our department. Thus, 20 patients met the inclusion criteria.

The included patients’ baseline characteristics are listed in Table 1.

**Table 1:** Baseline characteristics of the 20 included patients, intended to treat

	Unit	Mean (±SD)
Age	Years	66.0 (±6.5)
NIV settings		
BPM	per minute	12.9 (±2.5)
IPAP_min_	cm H_2_O	13.8 (±3.7)
IPAP_max_	cm H_2_O	17.5 (±3.3)
EPAP	cm H_2_O	5.4 (±0.7)
*T*_i_		1.3 (±0.2)
*V*_t_	mL	554.3 (±136.7)
PC/AVAPS	*n*	10/10
Lung function		
FEV_1_	L	0.67 (±0.44)
FEV_1_	% pred	27.8 (±16.2)
FVC	L	1.77 (±0.66)
MRC	Median (IQR)	4 (±1)
Pack years		44.6 (±18.8)
BMI	kg/m^2^	23.7 (±6.8)
*Sp*O_2_	%	93.4 (±3.1)
Oxygen supply	L	2.4 (±3.2)
Arterial gas values confirming AHRF		
pH		7.26 (±0.07)
*P*_a,CO_2__	kPa	11.3 (±1.8)
*P*_a,O_2__	kPa	8.5 (±1.7)
*st*BE	mmol/L	11.2 (±5.4)
Medication and therapy		
LAMA	*n* (%)	17 (85)
LABA	*n* (%)	18 (90)
ICS	*n* (%)	16 (80)
OCS	*n* (%)	4 (20)
Theophylline	*n* (%)	4 (20)
Rehabilitation	*n* (%)	11 (55)
LTOT	*n* (%)	18 (90)

NIV: non-invasive positive pressure ventilation; BPM: breaths per minute; IPAP: inspiratory positive airway pressure; EPAP: expiratory positive airway pressure; *T*_i_: inspiratory time; *V*_t_: ventilated volume per minute; PC: pressure control; AVAPS: average volume assured pressure ventilation; FEV_1_: forced expiratory volume in one second; FVC: forced vital capacity; MRC: Medical Research Council dyspnea score; IQR: interquartile range; BMI: body mass index; *Sp*O_2_: peripheral oxygen saturation; AHRF: acute hypercapnic respiratory failure; *P*_a,CO_2__: arterial carbon dioxide tension; *P*_a,O_2__: arterial oxygen tension; *st*BE: standard base-excess concentration in arterial blood; LAMA: long-acting muscarinic antagonists; LABA: long-acting β_2_ agonists; ICS: inhaled corticosteroids; OCS: oral corticosteroids; LTOT: long-term oxygen therapy.

Ten patients used the average volume assured pressure support (AVAPS) mode on the ventilator and ten patients used regular pressure controlled ventilation.

The patients were treated with a mean IPAPmax (maximum inspiratory positive airway pressure) of 17.7 cmH_2_O and a mean expiratory positive airway pressure of 5.4 cmH_2_O.

The variable ‘IPAPmin’ is the mean of the minimum IPAP settings in AVAPS mode.

Four of the 20 patients (20%) died during the 1-year follow-up.

Thus, we conducted the PP analysis on the remaining 16 patients. The results are listed in Table 2.

**Table 2:** Per protocol analysis on the 16 patients who were still alive 1 year after LTNIV initiation

Per protocol: variables at LTNIV initiation vs. after 1 year with LTNIV, *n*=16				

		Prior to LTNIV	After 1 year with LTNIV	
			
	Unit	Mean (±SD)	Mean (±SD)	*p*
FEV_1_	L	0.73 (±0.48)	0.71 (±0.44)	0.94
FEV_1_	% pred	30.7 (±16.7)	29.9 (±16.9)	0.93
FVC	L	1.77 (±0.66)	1.68 (±0.64)	0.78
LTOT	*n* (%)	14 (87.5)	11 (68.8)	0.20
pH		7.40 (±0.04)	7.42 (±0.03)	0.17
*P*_a,CO_2__	kPa	7.6 (±1.7)	6.7 (±1.4)	0.11
*P*_a,O_2__	kPa	9.0 (±2.1)	9.0 (±1.8)	0.98
*st*BE	mmol/L	7.4 (±5.5)	7.0 (±5.5)	0.76
AHRF admissions	*n*/year	2.4 (±0.6)	0.4 (±0.6)	<0.0001
Admissions	*n*/year	5.2 (±3.5)	1.9 (±3.2)	0.0092
Admission days	*n*/year	42.6 (±27.3)	11.9 (±22.3)	0.0015

LTNIV: long-term non-invasive positive pressure ventilation; FEV_1_: forced expiratory volume in one second; FVC: forced vital capacity; LTOT: long-term oxygen therapy; *P*_a,CO_2__: arterial carbon dioxide tension; *P*_a,O_2__: arterial oxygen tension; *st*BE: standard base-excess concentration in arterial blood; AHRF: acute hypercapnic respiratory failure.

**Fig. 1:**
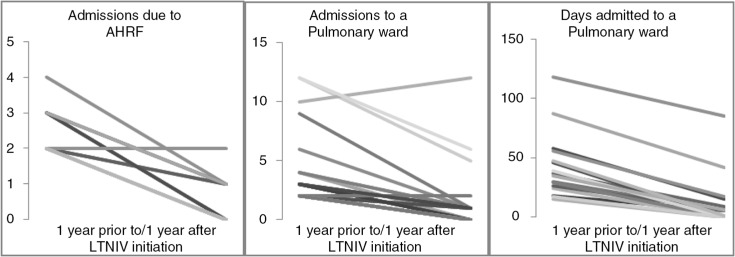
Number of admissions with AHRF, admissions to a pulmonary department *per se*, and days admitted for the 16 per protocol patients.

The patients’ mean number of AHRF episodes per year decreased from 2.44 to 0.44 (*p*<0.0001) (see Fig. 1).

Likewise, the mean number of admissions to a pulmonary ward decreased from 5.19 to 1.88 (*p*=0.0092).

The mean number of admission days decreased from 42.6 to 11.9 (*p*=0.0015).

No significant changes in arterial gases, lung function parameters, or LTOT status were found during the 1-year follow-up, although pH, *P*_a,CO_2__, and use of LTOT tended to decrease.

The patients tolerated the treatment well and used LTNIV for a minimum of 4 h a day.

## Discussion

In the present study, treatment with LTNIV reduced the number of recurrent AHRF, admissions, and admission time in a group of unstable COPD patients with multiple previous episodes of AHRF.

This is consistent with the results of Funk et al. and Cheung et al. (7, 8).

In accordance with previous studies, we found no significant changes in lung function following initiation of LTNIV (12–17).

As expected, arterial gases improved during the acute NIV treatment of AHRF during the index admission. In the following year, arterial gases tended to improve, though not significantly.

This finding corresponds with previous studies that have investigated the mechanism behind the effect of LTNIV in COPD patients with chronic respiratory failure (10, 11–18).

We found a 1-year mortality of 20% after LTNIV initiation. This is considerably lower than the 49.1% for COPD patients having survived an AHRF found by Chu et al. (4), although arguably the two studies’ settings and patients were not entirely comparable.

The 16 patients on which the PP analysis was based had a high compliance with LTNIV. This finding suggests a net gain in quality of life; the discomfort of LTNIV seems to have been outweighed by the stabilization of the disease with a reduction of admissions.

Were these positive effects merely caused by regression to the mean?

We are convinced that they are not and that the effects are attributable to LTNIV.

Despite not having a control group, we argue that this study was somewhat controlled, as the patients were their own controls:

the patients’ admission frequencies, lung functions, arterial gases, and so on prior to LTNIV initiation were compared with the same values after 1 year with LTNIV.

This assumption is reasonable, given the following:

- The patients’ general health was unlikely to improve over 1 year.
- In COPD treated according to GOLD, lung function does not improve spontaneously (19); it is more likely to deteriorate. We found no improvements in lung function.
- According to the ECLIPSE study, exacerbations of COPD are a great predictor of new exacerbations (20), and prior admissions due to exacerbations of COPD predict new hospitalizations due to exacerbation (21). However, this is somewhat contradicted by the finding that some patients with frequent exacerbations of COPD have a spontaneously declining rate of exacerbations (22).

Overall, we argue that the reduction of rates of admission due to exacerbations of COPD shown in this study should not occur absent a radical intervention such as LTNIV.

Theoretically, multiple mechanisms could attribute to the positive effects of LTNIV.

NIV relieves fatiguing respiratory muscles by reducing the end-expiratory lung volume and hence the degree of hyperinflation (3).

A reduction of hypercapnia, especially nocturnal, may lead to a slight alkalization of the patient's blood and an improvement of the sensitivity of the respiratory center to carbon dioxide (18–23).

Moreover, reduced levels of CO_2_ might reduce edemas in the airway walls (24).

In total, a reduction of hyperinflation and airway wall edema and an improvement of chemosensitivity should improve gas exchange (18–23). Theoretically, lung function could improve as well, though we and others have not been able to show this with significance.

Other mechanisms such as mobilization of sputum may be involved too: Positive end-expiratory pressure (PEEP) is known to help mobilizing mucus (25). As an advanced PEEP, LTNIV might help mobilize and clear excessive and mucous airway mucus in COPD. By clearing the mucus, bacterial colonies are removed, thereby reducing the risk of airway infections.

Should LTNIV not prevent exacerbations, it could be hypothesized that COPD patients who are treated with oral antibiotics, prednisolone, bronchodilators, and NIV at home simply do not deteriorate to a degree where their respiratory muscles fatigue and respiratory failure occurs. Although the number of exacerbations *per se* would remain unchanged, the number of severe exacerbations causing AHRF and admissions would still decrease as in the present study.

An analogy would be a bicycle rider on a very steep hill: At the steepest part, instead of becoming tired and crashing the bike, the rider is pushed by a scooter riding by, and thus he manages to ride over the hill.

NIV could be the push that allows the patient to get through the exacerbation without *crashing* – that is, developing fatigue, respiratory failure, and requiring hospital admission.

Whatever the mechanism of LTNIV in patients with severe, unstable COPD, the seemingly clear benefits should be investigated thoroughly in an RCT: the non-controlled design of this study is inferior to that of an RCT; the patients included with repeated episodes of AHRF were highly selected and not likely to be representative of all patients having survived an episode of AHRF due to COPD. Furthermore, the patients included had access to an increased level of care compared to the patients treated at our department – for example, the 24-h nurse-led phone hotline; this might have increased treatment compliance and thus improved the outcomes.

We acknowledge that the results presented are not necessarily entirely comparable to the results of other studies; however, we believe it is important to discuss the results of the study in the context of similar studies.

Optimally, an RCT of LTNIV for COPD patients with multiple previous episodes of AHRF is needed. Its focus should be mortality, admissions, exacerbations, quality of life, compliance, and a cost–benefit analysis.

However, we presume this RCT would be difficult to conduct, as the needed patients would be difficult to find and include.

Until investigated in an RCT, the results of the study suggest that LTNIV should be considered in patients with COPD and recurrent episodes of AHRF.

## Conclusions

The results of this study suggest that LTNIV should be considered for patients with COPD and repeated episodes of AHRF, as the treatment seemingly reduces the number of recurrent episodes of AHRF and admissions.

Further studies are desirable, though RCTs could be difficult to conduct.
